# Determination of abundance and symbiotic effectiveness of native rhizobia nodulating soybean and other legumes in Rwanda

**DOI:** 10.1002/pei3.10138

**Published:** 2024-03-19

**Authors:** Felix Nzeyimana, Richard N. Onwonga, Fredrick O. Ayuke, George N. Chemining'wa, Nsharwasi L. Nabahungu, Joseph Bigirimana, Umuhoza K. Noella Josiane

**Affiliations:** ^1^ Faculty of Agriculture University of Nairobi Nairobi Kenya; ^2^ Rwanda Agriculture and Animal Resources Development Board (RAB) Kigali Rwanda; ^3^ Rwanda Institute for Conservation Agriculture (RICA) Gashora Rwanda; ^4^ International Institute of Tropical Agriculture (IITA) Nairobi Kenya; ^5^ Department of Agriculture University of Technology and Art of Byumba Gicumbi‐ Rwanda; ^6^ College of Agriculture Animal Science and Veterinary Medicine, Crop Sciences Department University of Rwanda Kigali Rwanda

**Keywords:** aluminium toxicity, N‐fixation, plant nutrients, rhizobia abundance, soil acidity

## Abstract

Rhizobia diversity in the rhizosphere is one of the key promoters of biological nitrogen fixation between host legumes and microsymbionts, although related complex interaction may depend on various factors. This research was intended to assess the abundance of indigenous rhizobia isolates under various soil conditions, as well as their effectiveness to nodulate legumes such as soybeans. Factors such as soil properties and legume species influence the volume and symbiotic effectiveness of native rhizobia to nodulate crop legumes. To investigate the abundance of rhizobia isolates, legume crops were uprooted to obtain nodules for most probable number (MPN) determination of rhizobia isolates, and soybean (*Glycine max.*) was used to verify the presence of suitable and efficient rhizobia strains for nitrogen fixation. Soil samples were obtained from the holes out of which nodules were collected, and the laboratory analysis included pH, Mg, K, available P, organic C, Ca, and N to establish the correlation between the soil status and number of rhizobia isolates' cells. Significant variations (*p*‐value <.05) were observed in the cell counts of Rhizobia isolates from *Glycine max*, *Phaseolus vulgaris*, *Pisum sativum*, and *Vigna unguiculata*, particularly when compared to *Arachis hypogaea* isolates under acidic conditions. Notably, *Pisum sativum* and *Vigna unguiculata* showed consistent performance across all pH conditions. The number of rhizobia isolates was found to be significantly linked to total N and P deficiencies (*p* < .05). It was also established that total N was dependent on the number of rhizobia cells and that there is a strong correlation between organic carbon and N content. This study highlights the crucial role of understanding and optimizing conditions for rhizobia nodulation in diverse soil environments, emphasizing its potential impact on enhancing biological nitrogen fixation in legumes.

## Graphical abstract

Rhizobia diversity in the rhizosphere is one of the key drivers to enhancing biological nitrogen fixation between host legumes and microsymbionts, and the mutual complex interaction maybe governed by different factors. The aim of this research was to examine the abundance of indigenous rhizobia isolates under different soil conditions and their effectiveness in the nodulation of legumes such as soybeans. To investigate the abundance of rhizobia isolates, legume crops were uprooted to obtain nodules for most probable number (MPN) determination of rhizobia isolates, and soybean crop was used for authentication. In the same holes from where nodules were collected, soils were sampled for laboratory analysis to correlate the soil status and number of rhizobia isolate cells.

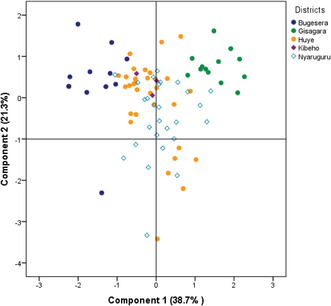

## 1 INTRODUCTION

Nitrogen fixation is a process in which rhizobia converts atmospheric dinitrogen (N2) into a form usable by leguminous plants (Masson‐Boivin & Sachs, 2018). Rhizobia microsymbionts are outfitted with the MoFe protein, the nitrogenase enzyme that allows for atmospheric N_2_ fixation into ammonia or nitrate form mostly absorbed by microorganisms (Downie, 2014). In specific instances, the inability of microbes to fix nitrogen results in the absence of root‐level nodulation in particular legumes. This highlights the importance of a well‐established symbiotic relationship between rhizobia and designated host plants, wherein successful linkage with root tissues occurs regardless of the abundance of microorganisms in the rhizosphere (Gamalero et al., 2022).

The presence of diverse rhizobia strains in the rhizosphere was noted to contribute differently to the symbiotic relationship with specific legume hosts (Pérez Carrascal et al., 2016). Preference for certain strains of *Rhizobium* involved in symbiosis with a host legume in various locations can be linked to soil characteristics—such as pH and plant nutrient availability as the latter underpin N‐fixation (Liu et al., 2014). In the mutually beneficial interaction involving legumes (plants) and microsymbionts (bacteria), the ensuing gains are attributed, albeit partially, to both the plant's genetic traits and the presence of the most suitable bacterial strain (Sachs et al., 2018). We can therefore assume that rhizobial diversity is definitely forming the foundational basis for the observed mutual advantages, and subsequently, to crop productivity (Barrett et al., 2015). Nodulation occurs in situations allowing for the legumes to select appropriate rhizobia from various nitrogen‐fixing microbial types, but in a given microbial community under different soil conditions, some strains may alternatively contribute to functions such as nitrogen fixation, nutrient cycling, or other roles when interacting with diverse legume crops (Nohwar et al., 2019). Having a legume crop inoculated with the suitable rhizobia strain leads to significant [positive] changes in the plant, by way of physical and chemical processes allowing for energy intake and conversion of atmospheric nitrogen into ammonia through the plant‐bacteria interaction (Moreau et al., 2019).

In the process of biological nitrogen fixation, rhizobia are with over a hundred bacteria making up 12 genera comprised of—among others—Alpha‐ and Beta‐proteobacteria; this indicates that rhizobia are most frequently found in symbiotic associations with legume crops (Peix et al., 2014). Legumes are well‐suited for actively participating in nitrogen fixation through soil microsymbionts and the bonding is very precise for each type of rhizobium species in such a way that the bacteria only associate with a specific legume cultivar . It is common knowledge that changes in environmental factors—including pH, soil nutritional status, and salinity—may have a significant impact on effective symbiosis occurrences (Alves et al., 2021). All of the elements involved in nodulation and N‐fixation, from rhizobia survival and growth to infection and nodulation, and mutualistic benefits, depend on the availability of nutrients like nitrogen (N) and phosphorus (P). The research sought to evaluate the presence of native rhizobia isolates in different soil environments and their capacity to form nodules on legumes, particularly soybeans. The research offers valuable insights into the potential contributions of indigenous rhizobia to the symbiotic relationships with legumes.

## 2 MATERIALS AND METHODS

### 2.1 Site description

**FIGURE 1:**
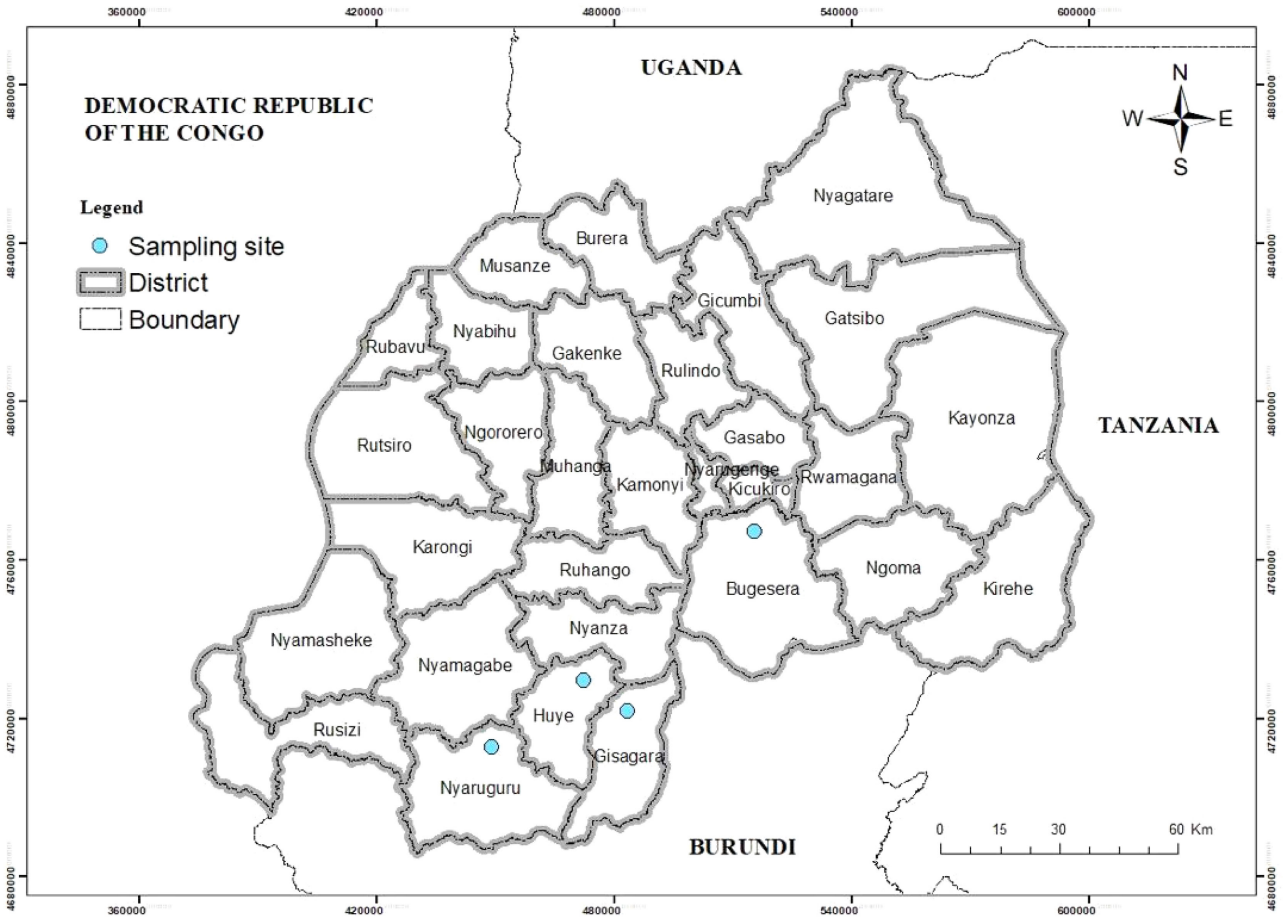
Location of districts (with big dots) where nodules were collected (Adapted from Ministry of Local Government and Social Affairs (MINALOC).

The study sites included Bugesera, Huye, Nyaruguru and Gisagara districts. These locations varied in terms of soil types and conditions, with diverse rainfall patterns where Bugesera: average annual rainfall of 800 mm, Huye: average annual rainfall of 1300 mm, in the Eastern part of Nyaruguru: average annual rainfall of 1200 mm, Kibeho located in the central of Nyaruguru district with average annual rainfall of 1400 mm, Gisagara: average annual rainfall of 1100 mm, and temperatures of all districts with average annual temperature ranging from 18°C to 22°C (Figure 1).

Bugesera, Huye, Nyaruguru, and Gisagara districts were selected due to their diverse soil acidity levels and conditions, providing a representative sample that allows for a comprehensive assessment of rhizobia performance across varying agroecosystems. The socio‐economic activities in these districts of Rwanda primarily revolve around subsistence agriculture, including crop cultivation and animal husbandry, with efforts to improve infrastructure and access to education and services.

### 2.2 Rhizobia population sampling

The study on legume crops considered several factors to ensure a comprehensive representation of the rhizobia population. First, the study considered the concept of sector‐level farmer typology based on the socio‐economic characteristics of farmer households. This approach acknowledged the inherent variability in farming practices and resources among different sectors of the population, such as poor, medium, and rich farmers. By including representatives from each sector, the study aimed to capture a wide range of perspectives and practices in legume cultivation. Furthermore, the research ensured that the sample size was adequate to detect a significant effect of calculated size, following the formula set forth by Kothari (2004), which provided a systematic approach to determine the appropriate sample size based on parameters such as the level of precision desired and the variability within the population.

(1) $$\text{Population sample}\;n=\frac{z^{2}\cdot p\cdot q}{e^{2}}=\frac{{\left(1.96\right)}^{2}*0.5*0.5}{{\left(0.1\right)}^{2}}=96$$

where *p* = population reliability (or frequency estimated for a sample of size *n*) equals 0.5; *q* = 1 – *p*; *z* = confidence level (1.96); *e* = margin of error (10%) and *n* = size of sample.

This ensured that the sample selected for the study was large enough to provide reliable and meaningful findings while considering the available resources and constraints. By employing these rigorous sampling techniques, the study aimed to enhance the generalizability of the findings. The implementation of random sampling was achieved in the field by systematically choosing random sites in the district to select specific locations for sampling. This meticulous process ensured that each legume plant had an equal chance of being chosen, thereby minimizing the potential for systematic errors. The farm typology approach was chosen to collect nodules, aiming to encompass diverse agricultural practices and environmental conditions for a representative sample. This categorization of farmers allows for a comprehensive exploration of how various factors influence legume nodulation. The combined use of these sampling methods provided a comprehensive and representative sample of legume crops and farmers, enabling the study to draw robust conclusions and make relevant recommendations for legume crops and nutrients availability relationship.

### 2.3 Nodule collection

Nodules were collected from various legume crops (*Arachis hypogaea*, *Glycine max*, *Phaseolus vulgaris*, *Pisum sativum* and *Vigna unguiculata*) and places (Bugesera, Huye, Nyaruguru, and Gisagara districts) across Rwanda, especially targeting plants at flowering stage. Ten nodules for each selected legume crop were kept in McCartney's glass bottles using silicate gel to ensure optimal conditions for adequate conservation and long‐term storage of rhizobia nodules (Sahgal & Jaggi, 2018). The study aimed to assess the soil characteristics and the abundance of rhizobia bacteria associated with legume crops in a specific region. In the study, we employed purposive sampling to target non‐inoculated legume crops, considering factors such as plant size and growth stage to capture active rhizobia, and geographical location to account for varying acidity levels in the soil. This intentional selection aimed to explore the natural characteristics and interactions of legumes without prior inoculation, providing valuable perspectives into their symbiotic relationships and soil conditions. Soil samples were collected from the rhizosphere at 30 cm of these legume plants to analyze soil pH, nutrient levels, and organic carbon content. About 500 g of soil samples were taken from each of the locations where plants were uprooted. A total of 500 g were separated for analysis, with 250 grams allocated for soil most probable number (MPN) and the remaining 250 g designated for soil analysis. This division allowed for a comprehensive examination of both microbial presence through MPN and physico‐chemical properties. Grown non‐inoculated legume crops were targeted in fields for nodules sampling. Additionally, nodules from the legume roots were sampled to evaluate the presence and abundance of rhizobia bacteria. This approach provided valuable insights into the soil status and rhizobia populations associated with legume crops in the targeted region, contributing to a better understanding of the symbiotic relationship between legumes and rhizobia under different soil characteristics. The isolation of nodules on a pure culture using agar yeast mannitol (AYM) extraction involved a series of steps to obtain uncontaminated bacterial strains, specifically Rhizobium, from legume root nodules. Initially, healthy nodules were collected and subjected to surface sterilization to minimize external contaminants. The nodules were then dissected or crushed under sterile conditions. AYM agar medium, comprising yeast extract, mannitol, agar‐agar, and essential nutrients, were prepared and autoclaved for sterilization. The nodule tissues were inoculated onto AYM agar plates, and following an incubation period, individual bacterial colonies were developed (Malik, 1992), using *Congo red*, whereby only rhizobia were morphologically identified from other microorganisms.

### 2.4 Seed surface sterilization and germination

Seeds were sterilized on the surface using 10% of hydrogen peroxide solutions for 20 min, and hard‐coated seeds were appropriately handled to avoid contamination and ensure rigorous test of microbial activity. Seed viability selection singled out only materials of which respective germination rates were over 85% and all the treated seeds were rinsed using distilled water prior to being wiped dry with clean white paper. The seeds were initially arranged on paper, followed by a rinsing step with distilled water. Subsequently, they were transferred to sterile Petri dishes, and placed on surfaces that had been rendered sterile. Each petri dish contained 20 to 30 seeds, which were germinated in plates. Two milliliters of distilled water per day was added for a duration of 7 days, and the seeds were ensured adequate light exposure throughout the process.

### 2.5 Serial dilution preparation

About 100 g soil sample was collected and diluted in 900 mL of N‐free solution (294.1 g/L of Cacl_2_.2H_2_O; 136 g/L of KH_2_PO_4_; 6.7 g of Fe‐citrate; 123.3 g of MgSO_4_.7H_2_O; 87 g of K_2_SO_4_; 0.338 g of MnSO_4_.H_2_O; 0.247 g of H_3_BO_3_; 0.288 g of ZnSO_4_.7H_2_O; 0.1 g CuSO_4_.5H_2_O; 0.056 g CoSO_4_.7H_2_O; 0.048 g Na_2_MoO_2_.2H_2_O) (Broughton & Dilworth, 1970), then kept on shaker for 3–7 days and removed afterwards depending on the desired growth periods. Serial dilutions of the broth culture were made for all the strains with two replications. Six tubes were set with 9 mL of autoclaved distilled water. One milliliter of the broth culture was taken through a tenfold (10^−1^ to 10^−6^) dilution. The broth culture was aspirated using a 1‐ml sterilized graduated pipette and immediately poured into Tube 1. In the first tube, the content was mixed 5 times prior to being transferred into another tube. The same process was carried out in successive tubes and a new pipette was picked and sterilized before utilization at each stage. In addition, a new sterilized pipette was used for one strain and one dilution, starting with the highest numbered dilution, setswise. For a brief moment, the pipettes to be used were sterilized using a Bunsen burner flame to avoid contaminating the following tube (Vincent, 1970).

(2) $$\text{Calculation of dilutions}=\frac{\text{Volume of sample}}{\text{Volume of sample}+\text{Volume of diluent}}$$

### 2.6 Plants infection count

MPN determined the number of strains effectively working with the tested legume, as well as the rhizobia strain(s) with a potential to nodulate the host plant. The SB24 soybean variety was chosen as a test crop for the soil MPN process, and the very same variety was tested to gauge the seed germination level. The legume used in implementing the MPN method is considered suitable as it belongs to the same group of legumes with the likelihood of nodulating with rhizobia. Polypropylene pouches of 16 × 18 cm with liner wick leaves were autoclaved while covered with aluminum papers. Racks—made up of 14‐gauge steel wires and a wooden board on which the said wires were attached to carry the pouches—were built on the basis of 1.5 cm spacing between metal frames. Plant nutrients' solutions were taken through autoclave sterilization and each growth pouch received 30 mL of the solution before seed plantation. It is worth noting that each rack was carrying 45–50 growth pouches.

To detect contamination, dilution's sets from the non‐inoculated control treatments were established and injection was done from the largest to the smallest dilutions for optimal control of any contamination that may have occurred during nodulation involving the highest dilutions. Seeds with about 1.5 cm radical length size were selected and transferred—under aseptic conditions—to designated pouches, on a “one‐seed‐per‐pouch” basis, then placed into a small hole made in the hollow of the wick liner. Sixty pouches per strain were set, to allow for dilutions of 10^−1^–10^−6^ and 2‐time replications.One milliliters of each inoculated dilution (from 10^−1^ to 10^−6^) was pipetted into two replicates for each strain—also comprising the control. Inoculation was conducted, starting from the highest dilutions' aliquots to the lowest successions, using identical pipettes.

Plants were checked on a daily basis to prevent the pouches from slanting. The surveyed pouches were refilled with a nutrient solution when necessary. In most cases, nodule formation was spotted 2 weeks after planting, the latest occurrence having been observed 21 days after planting. For records' purposes, the presence of nodule(s) was marked with a positive (+) sign (the number of nodules being of no significance or relevance to the sign), while a negative (−) stood for “No nodule formation observed”.

### 2.7 MPN determination

Six dilution steps (*S* = 6) with two replications (*n* = 2) were used in the experiment to calculate the MPN of rhizobia. Upon listing of “positives” and “negatives” records, the sum of the positives' records from two replicates in all the strain's dilutions was calculated considering nodulated units, and then the total number of nodulated units from all dilution steps was calculated (m). MPN per gram of soil (X) was calculated using the tenfold method suggested by Vincent (1970).

$$X=\frac{m\times d}{V}$$

where m = No from the MPN table (Tenfold) resulted from the total No of nodulated units. d = Lowest dilution (10–1). v = Applied volume of aliquot during inoculation (1 mL).

### 2.8 Statistical analysis

The soil physico‐chemical properties, that is, soil pH, Mg, K, Average P, Organic C, Calcium, and total N were averaged for all the sites surveyed. Additionally, the total number of nodulated units per gram of soil was log‐transformed prior to the analysis. Furthermore, the data was checked for normality and equality of variance assumptions before being subjected to the analysis of variance test using mixed models where sites were a random factor and plant species a fixed factor. Treatment means were separated using Tukey's test at *p* = .05. We also used the stepwise multiple regression analysis to assess the influence of soil physico‐chemical properties (pH, Mg, Al^3+^, Organic C, Ca, total N, Available P, H^+^, and Cations Exchange Capacity (CEC)) on the total number of nodulated units using the following model:

$$Y=\alpha +\beta_{1}X_{1}+\beta_{2}X_{2+}\;\beta_{3}X_{3+}\;\beta_{4}X_{4}+\beta_{5}X_{5}+\beta_{6}X_{6}+\beta_{7}X_{7}+\beta_{8}X_{8}+\beta_{9}X_{9}+\beta_{10}X_{10}+Ɛ,$$

Where *Y* is total number of nodulated units per g of soils, α: the intercept, β_1_ the coefficient of pH (X_1_)_,_ β_2_ the coefficient of Mg (X_2_), β_3_ the coefficient of Al^3+^(X_3_), β_4_ the coefficient of Organic C (X_4_), β_5_ the coefficient of Ca (X_5_), β_6_ the coefficient of total N (X_6_), β_7_ the coefficient of Available P (X_7_), β_8_ the coefficient of H^+^ (X_8_), β_9_ the coefficient of Cations Exchange Capacity (X_9_) and Ɛ is the error term. Model selection was done using the Bayesian Information Criterion and the model with the smallest (BIC) was selected. The correlation analysis was performed for all the variables in the study. Given the variables were interrelated, we had an idea to identify the ones that could explain the most variations. To this end, we used Bartlett's test of sphericity to test the null hypothesis that the correlations among variables were zero or that the correlation matrix was an identity matrix (Raykov & Marcoulides, 2008) for all the physico‐chemical properties and the total number of nodulated units per gram of soils.

The results of Bartlett's test of sphericity indicated that the test was significant (*χ* ^2^ = 593.511, df = 10, *p* < .000), Furthermore, the results of Kaiser‐Meyer‐Olkin (KMO) measure with regard to sampling adequacy was 0.718, also confirming the relationships between the study variables and their appropriateness for Principal Component Analysis (PCA). The data set was then subjected to PCA and the relationship between the soil physico‐chemical properties, and the total number of nodulated units per gram of soils along with the sites was visualized using the PCA biplots. Statistical analyses were conducted using Genstat Software (19th Edition). pH variation was statistically analyzed using UNIANOVA with a factorial design involving “Sites” and “Rhizobia isolates from legume crops.” Post hoc LSD tests, estimated marginal means (EMMEANS), and tables of adjusted comparisons for Sites, Rhizobia isolates, and their interaction were computed. The analysis indicated a significant relationship between Sites and pH variations at a significance level of *α* = 0.05.

## 3 RESULTS

### 3.1 Soil's physico‐chemical properties at study sites

The results indicated that rhizobia cell number in log of 10 ranged from 0.00 to 5.85 with a mean of 5.845 ± 0.014 (Table 1). Soils were extremely acidic to slightly alkaline, with pH varying from 4.19 to 7.56, and most soils were moderately acidic with a mean pH of 5.83 ± 0.595. The soil Mg in the sites where soil samples were collected was low to high, with values ranging from 0.16 to 0.52. Most sites had a relatively medium level of Mg with a mean of 0.67 ± 0.068. As part of the organic matter, the recorded organic C was 1.83 ± 0.187 (Mean ± SE) and this ranged from low to high of between 0.66 and 3.61. The total N ranged from 0.01 to 0.21 (very low to moderate), with a mean of 0.08 ± 0.008 (Mean ± SE). Available P—a key element determining underlying crop yields—was measured at 18.66 ± 1.904 (Mean ± SE), with values ranging from 4.59 to 103.66 (very low to high).

**TABLE 1:** Soil's physico‐chemical properties at study sites.

	Unit	Mean	Range
Rhizobia cell number	Log10	1.51	0.0–5.85
pH		5.8	4.2–7.6
Mg	mg kg^−1^	0.7	0.2–1.8
K	mg kg^−1^	0.5	0.03–2.3
Av. P	mg kg^−1^	18.7	4.6–103.7
Org. C	%	1.8	0.7–3.7
Ca	%	1.9	0.5–4.7
Total. N	%	0.1	0.0–0.2

### 3.2 Rhizobia isolate comparison among legume crops under soil conditions

The Rhizobia Isolates (Table 2) involving *Glycine max*, *Phaseolus vulgaris*, *Pisum sativum*, and *Vigna unguiculata* exhibited significant margins (*p*‐value < .05) with *Arachis hypogaea's* isolates under acidic conditions. The results revealed no significant differences (*p*‐value > .05) in rhizobia isolates of *Glycine max*, *Phaseolus vulgaris*, *Pisum sativum*, and *Vigna unguiculata* under soil acidity conditions.

**TABLE 2:** pH variations, highlighting the relationship between various “Rhizobia isolates from different legume crops” and pH levels, focusing on main effects and interaction, and marking a significant association at a significance level of *α* = 0.05.

Rhizobia isolates		Mean difference (I–J)	Std. error	Sig.^d^
*Arachis hypogaea*	*Glycine max*	−0.711^a^ ^,^ ^b^	0.3	0.025
	*Phaseolus vulgaris*	−0.868^a^ ^,^ ^b^	0.3	0.004
	*Pisum sativum*	−0.787^a^ ^,^ ^b^ ^,^ ^c^	0.3	0.015
	*Vigna unguiculata*	−1.117^a^ ^,^ ^b^ ^,^ ^c^	0.4	0.002
*Glycine max*	*Arachis hypogaea*	0.711^a^ ^,^ ^c^	0.3	0.025
	*Phaseolus vulgaris*	−0.157	0.2	0.390
	*Pisum sativum*	−0.076^c^	0.2	0.726
	*Vigna unguiculata*	−0.406^c^	0.3	0.138
*Phaseolus vulgaris*	*Arachis hypogaea*	0.868^a^ ^,^ ^c^	0.3	0.004
	*Glycine max*	0.157	0.2	0.390
	*Pisum sativum*	0.082^c^	0.2	0.670
	*Vigna unguiculata*	−0.248^c^	0.3	0.327
*Pisum sativum*	*Arachis hypogaea*	0.787^a^ ^,^ ^b^ ^,^ ^c^	0.3	0.015
	*Glycine max*	0.076^b^	0.2	0.726
	*Phaseolus vulgaris*	−0.082^b^	0.2	0.670
	*Vigna unguiculata*	−0.330^b^ ^,^ ^c^	0.3	0.237
*Vigna unguiculata*	*Arachis hypogaea*	1.117^a^ ^,^ ^b^ ^,^ ^c^	0.4	0.002
	*Glycine max*	0.406^b^	0.3	0.138
	*Phaseolus vulgaris*	0.248^b^	0.3	0.327
	*Pisum sativum*	0.330^b^ ^,^ ^c^	0.3	0.237

*Note*: Based on estimated marginal means.

^a^ The mean difference is significant at *p* < .05.

^b^ An estimate of the modified population marginal mean (I).

^c^ An estimate of the modified population marginal mean (J).

^d^ Adjustment for multiple comparisons: Least Significant Difference.

### 3.3 Prediction of Rhizobia cell number using the soil physico‐chemical properties

The relationship between the soil's physico‐chemical properties and the total number of nodulated units per g of soil Rhizobia cell number is presented in Table 3. Based on the study's data, the best way to predict Rhizobia cell number was to carry out a stepwise regression analysis. Out of 10 independent variables considered in the analysis (pH, Mg, Al^3+^, Organic C, Ca, total N, Available P, H^+^), and Cations Exchange Capacity (CEC), only two variables (available P and total N) happened to significantly influence the rhizobia cell number. Increasing total N was associated with increasing rhizobia cell number while available P was negatively related to rhizobia cell number. From this study's results, it seems that rhizobia cell number could be better predicted by Equation Y (rhizobia cell number) = 71.29 + 167.47 Total N – 0.22 available P. While the intercept was highly significant, estimates of the remaining variables were significantly related to rhizobia cell number (*p* < .05).

**TABLE 3:** Stepwise multiple regression results using soil physico‐chemical properties as predictors for rhizobia cell number (rhizobia cell number).

Variables	Para. Estimates	Standard errors	*p*‐values
Constant	71.29	5.297	.000***
Total nitrogen (%)	167.47	83.037	.021*
Available phosphorus	−0.22	0.119	.026*

* Significant at the 0.05 level.

*** High significant at the 0.001 level or lower.

### 3.4 Correlations between soil's physico‐chemical properties and Rhizobia cell number

The Pearson's correlation coefficients between soil's physico‐chemical properties and the total number of nodulated units per unit per g of soil rhizobia cell number are presented in Table 4. Most correlation coefficients among the study parameters were strong, positive, and highly significant (*p* < .01). A negatively strong and highly significant correlation was recorded between Al^3+^ and pH. Other correlation coefficients, such as the ones between K and pH, organic C and Mg, Ca and K, and available P and pH were positively and highly significant. However, the correlations between Al^3+^ and Mg and between Ca and Al^3+^ were highly significant. Overall, most correlation coefficients between soil's physico‐chemical properties and rhizobia cell number were positively significant.

**TABLE 4:** Pearson's correlation coefficients between soil's physico‐chemical properties and rhizobia cell number.

	Rh. Log nr.	pH	Mg	Al3+	K	Org. C	Ca	Total. N	Av. P
Rh. log nr.	1								
pH	−0.21*	1							
Mg	0.06^ns^	0.48**	1						
Al^3+^	0.17^ns^	−0.60**	−0.38**	1					
K	0.08^ns^	0.34**	0.42**	−0.20^ns^	1				
Org. C	0.08^ns^	0.07^ns^	0.47**	0.08^ns^	0.40**	1			
Ca	−0.04^ns^	0.61**	0.75**	−0.35**	0.45**	0.44**	1		
Total. N	0.08*	0.92**	0.16^ns^	0.22*	0.31*	0.78**	0.22*	1.	
Av. P	−0.10^ns^	0.47**	0.31**	−0.12^ns^	0.38**	0.32**	0.40**	0.29**	1

*Note*: As for PCA's results, they showed four principal components, altogether corroborating 80.4% of the data variability (Table 5). The first principal component (PC) ascertained 38.7% of the data variability, while the second and the third asserted 21.3% and 11.2% respectively.

Abbreviation: ns, non‐significant.

* Correlation is significant at *p* < .05.

** Correlation is highly significant at *p* < .01.

**TABLE 5:** Variance totals asserting respective cumulative percentages.

Component	Initial eigenvalues			Extraction sums of squared loadings		
	Total	% of variance	Cumulative %	Total	% of variance	Cumulative %
1	3.486	38.733	38.733	3.486	38.733	38.733
2	1.914	21.265	59.999	1.914	21.265	59.999
3	1.005	11.170	71.168	1.005	11.170	71.168
4	0.751	8.342	79.511			
5	0.617	6.852	86.362			
6	0.546	6.062	92.424			
7	0.347	3.854	96.278			
8	0.201	2.228	98.506			
9	0.134	1.494	100.000			

Rhizobia cell number were positively associated with PC 3; pH, Available P., Ca, Mg and K were positively associated with PC 1 while Al^3+^, Total N and Organic C. were positively associated with PC 2. To enhance the interpretability of the principal components, orthogonal rotation was conducted. The results of the orthogonal rotation indicated that each variable loaded highly on one component and low on the other components. Rhizobia cell number (Table 6).

**TABLE 6:** Variables' loadings to the three principal components and the high loading for each variable are indicated in bold.

	Component		
	1	2	3
Log no. rhizobia	−.117	0.362	0.815
pH water	.695	−0.520	−0.057
Al3 + (meq/100 g)	−0.438	0.675	−0.146
Total.N (%)	0.445	0.766	−0.185
Org.C (%)	0.637	0.660	−0.002
Av.P (ppm)	0.630	0.048	−0.335
Ca (meq/100 g)	0.845	−0.110	0.220
Mg(meq/100 g)	0.794	−0.093	0.327
K(meq/100 g)	0.672	0.110	−0.119

**FIGURE 2:**
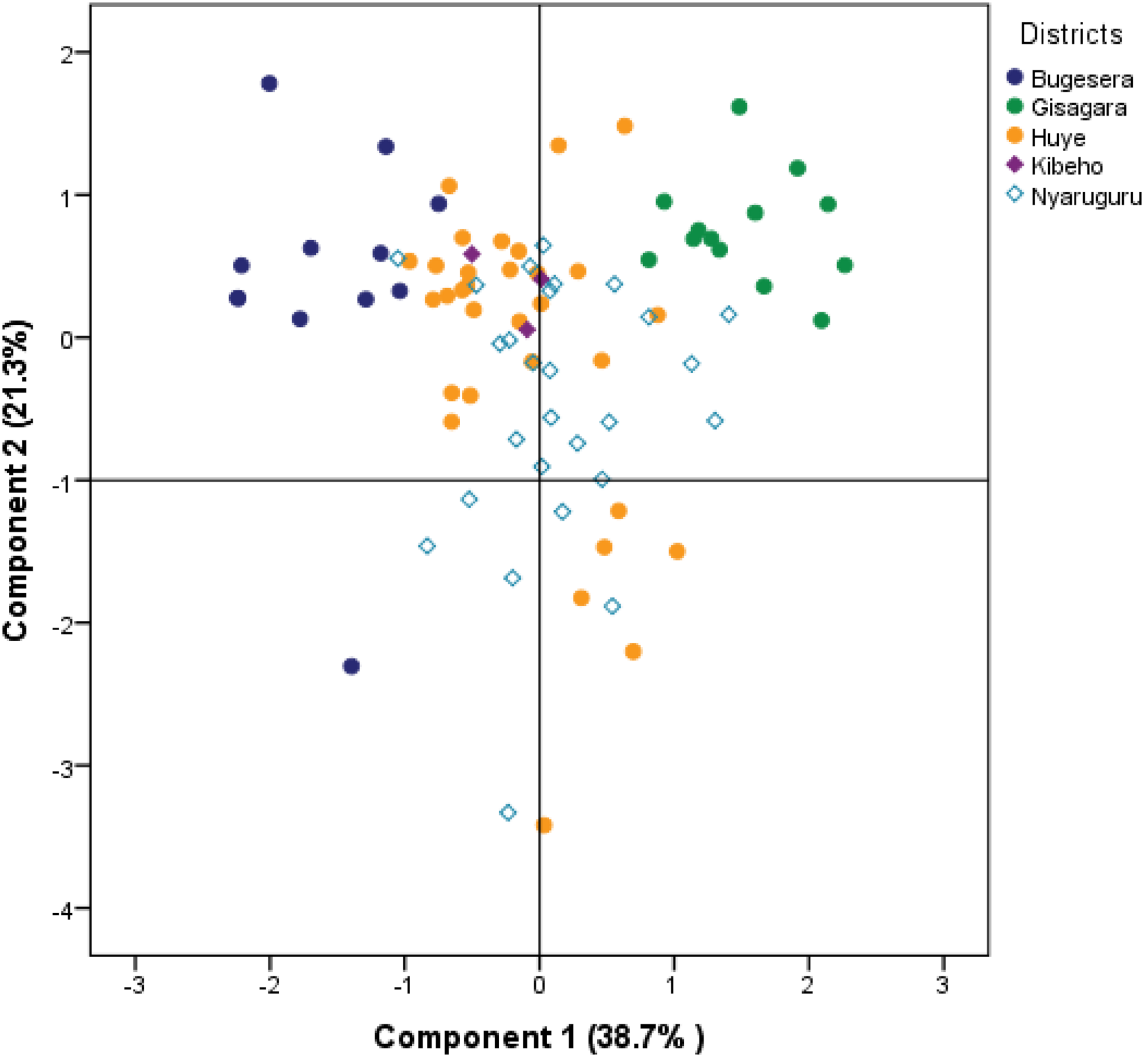
Biplot illustrating the results of principal component analysis conducted on 86 soil samples collected from diverse study sites following legume crop nodule collection. The biplot displays the distribution of isolates across different sites, highlighting their relationships in relation to soil status.

The results of PCA analysis for both the rhizobia cell number and the soil's physico‐chemical properties were displayed using a biplot (Figure 2). The biplot accounted for 95.5% of the data variations, out of which 38.7% were from the first principal component and 21.3% stemmed from the second principal component. Ca, Mg, and pH significantly influenced the principal component 1, while Al^3+^, Total N, and Organic C. influenced the principal component 2. The variables that were highly correlated include Ca, Mg, and pH; and total N and organic C judging by the smallness of the angle between the vectors representing those variables. On the other hand, variables such as available P and rhizobia cell number or organic C seemed not to be related considering a close‐to‐a‐right angle between the vectors representing these variables. The data points representing variables such as Al^3+^ and available P or Al^3+^ and Ca diverge and form a large angle, indicating that the variables they represent are negatively correlated. These results imply that fewer parameters, such as available P, total N, and Ca could be used to predict the number of rhizobia isolates, an important element to improve legume crops' yields.

## 4 DISCUSSION

This study (Tables 1 and 2) highlighted significant variations in pH levels, particularly the relationship between diverse “Rhizobia isolates from different legume crops” and pH, within the context of the soil's physico‐chemical properties at the study sites. The relationship between pH and soil properties significantly influences “Rhizobia isolates from different legume crops,” showcasing a dynamic relationship where the characteristics of the soil impact the existence of these microorganisms (Ohyama, 2017). Soil microbial communities and nutrient cycling have been emphasized in sustaining rhizobia populations, highlighting their importance in promoting rhizobia abundance in legume crops (Chamkhi et al., 2022).

The results (Table 2) pointed out the presence of rhizobia strain under very acidic condition, also showing that it did not affect the host plant's growth and that of the microsymbiont. Some rhizobia strains were found to survive and function under highly acidic conditions with a pH below 4, despite their preference for slightly acidic to neutral pH ranges (De Meyer et al., 2016). Although acidity was a hindrance to the growth of certain bacteria in the soil, it is evident that inoculants containing resistant native strains have received little attention in the agricultural sector, hence the need to work on inoculants with acid‐tolerant strains while using other types under normal conditions (Atieno & Lesueur, 2019). The mutualistic relationship between plants and rhizobia involves nitrogen fixation, but it can be influenced by soil acidity. Acidic conditions negatively influence both plants and rhizobia, hindering plant growth and the survival and colonization of rhizobia on plant roots (Shankar et al., 2021). Neutral pH is more favourable, supporting optimal nutrient availability, plant growth, and microbial N‐fixation (O'Callaghan et al., 2022). However, certain acid‐tolerant rhizobia may still establish symbiotic relationships with plants under acidic conditions (Ferguson et al., 2019), and some legume crops exhibit resistance to acidic soil, enabling them to tolerate and thrive in such environments (Li et al., 2023).

The results (Figure 2) also revealed slight differences in the number of Rhizobium cells in nodules taken from five different legume crops, depending on location and legume isolates, and showed that the five isolates differed from each other, as per the type of soil and the agro‐ecological zone. In addition, research studies investigated the variation in rhizobia's cell numbers across different agro‐ecological zones. Significant differences in rhizobia cell numbers among various regions have been observed, suggesting the influence of environmental factors on the distribution and abundance of rhizobia populations (Herridge et al., 2008). Moreover, variations in rhizobia populations across different agro‐ecological zones were linked to soil characteristics and climatic conditions (Jaiswal et al., 2016).

Considering the locations and rhizobia isolates from where legume crops were collected, the soil's acidity had a notable impact on the rhizobia population concerning host plants. Specifically, isolates from Arachis hypogaea were less abundant under acidic conditions compared to other legume crops. The types of rhizobial communities could be determined according to soil acidity level, where *Rhizobium tropici* are found in acid soils, *MesoRhizobium* spp. at moderate acidity and *SinoRhizobium* spp. are adapted to alkalinity conditions (Bala & Giller, 2006). Notwithstanding the above statement, the fact remains that several adaptive physiological mechanisms allow some legume crops to remain and nodulate with elite rhizobia strains under low acidity conditions (Choudhary et al., 2018).

From this study (Table 3), it is obvious that the occurrence of rhizobia populations in the rhizosphere depends on nitrogen and phosphorus content in the soil. A given level of Nitrogen content was positively correlated to the number of rhizobia cells, and the availability of phosphorus has also been found to positively affect N‐fixation potential in legume crops. Previous studies have shown that legume crops with microbial bacteria can convey to the soil up to 70% of its N‐fixation‐related demand (Santachiara et al., 2018), although it is worth pointing out that high N content limits the fixation of atmospheric nitrogen in some legume cultivars (Santachiara et al., 2019). It is equally important to work on the phosphorus use efficiency to improve the N_2_ fixation, as the same nutrient contributes to root system development, to plant nutrient availability, and, altogether, to the legume crop growth (Mitran et al., 2018).

In the frame of this study, the Pearson's correlation test exhibited the negative impact of one or more soil parameters on others. It also came out that aluminium toxicity was negatively linked with soil acidity and the presence of magnesium (Mg). In addressing the soil acidity problem, extraction of Al^3+^ decreases whereas Ca, K, Mg, and P concentrations are extracted in great amounts (Fung & Wong, 2002). Results revealed a negative correlation between Al^3+^ and other soil parameters such as pH, Mg, and Ca^2+^, except where total Nitrogen was available in the presence of aluminium toxicity. According to previous studies, exchangeable Ca^2+^ and Mg^2+^ are boosted when soil acidity is corrected, and an antagonistic trend is observed with the disappearance of exchangeable Al^3+^ (Han et al., 2019). Soil amendments correct the acidity and aluminium toxicity, but acid‐tolerant microbial environment is not disturbed by the presence of aluminium (Violante et al., 2010) due to nutrients—such as Ca and Mg which impede the Al action–, as it also allows available Nitrogen and P to enrich the soil in the presence of Al^3+^ (Rahman et al., 2018).

The results (Table 6) revealed a correlation between variables, where nutrients like Mg, organic C, Ca, N, and CEC exist under good pH levels. Negative correlations of pH, Mg, and Ca were present when there is a positive correlation between Al and N. They also show that Increased carbon storage enhances the CEC, Ca^2+^, and Mg^2+^ (Ramos et al., 2018) and that the C/N ratio greatly contributes to building soil carbon reservoir (Cotrufo et al., 2019). In addition, it was also found that soil acidity negatively affects crop nutrients availability and helps mobilize aluminium in soil (SHI et al., 2019). In terms of reactiveness, it came out that soil pH exhibited a positive response to the collective presence of N, P, K, and organic carbon. In the same vein, it was observed that Nitrogen content in soil was related to the amount of rhizobial microbes. It is worth pointing out that previous studies have demonstrated that—coupled with microbial activity—soil pH influences soil's essential nutrients, thus enhancing N‐fixation (Kang et al., 2021).

## 5 CONCLUSION

The study confirmed the negative effect of soil acidity on nutrient availability and how, in turn, poor soils disadvantage the existence of rhizobia population. Furthermore, some of the rhizobia isolates nodulated with their legume hosts under acidic condition and got rid of the negative effect of aluminium toxicity, thereby leading to significant N‐fixation. The results revealed the positive contribution of optimal pH on plant nutrients such as N, P, K, Mg, org. C, Ca, and CEC and on rhizobia abundance.

This study suggests that the introduction of indigenous rhizobia strains, used in normal environmental conditions, would be the best way to increase the rhizobia abundances. Under acidic conditions, most plant nutrients are unavailable, and this situation will require correcting the pH by applying soil amendments such as lime and organic manure to ameliorate soil acidity and enhance nutrient availability. Furthermore, rhizobia strains with the potential to withstand acidic conditions need to be promoted to overcome the N‐fixation problem where soil acidity has happened to be a concern. Aluminium toxicity negatively affects the availability of crop nutrients under acidic conditions, but some of the host plants with their microsymbionts can produce the N‐fixation under low pH conditions.

### CONFLICT OF INTEREST STATEMENT

Mr. Felix Nzeyimana, Dr. Fredrick O. Ayuke, Prof. George N. Chemining'wa, Prof. Richard N. Onwonga, Dr. Nsharwasi L. Nabahungu, Dr. Joseph Bigirimana and Dr. Umuhoza K. Noella Josiane declare that they have no conflict of interest.

## FUNDING INFORMATION

This research received funding from USAID through Michigan State University and Borlaug Higher Education for Agricultural Research and Development Program.

## Supporting information


Supporting Information S1.

## Data Availability

The data supporting this study will be shared upon worthy request to the corresponding author.

## APPENDIX A

### A.1

Table A1.

**TABLE A1:** Number (m) of rhizobia estimated by the plant infection count (after Vincent, 1970). C. 10‐fold dilutions (A = 10)^a^.

Positive tubes			Dilution steps (s)		
*n* = 4	*n* = 2	*s* = 10			
40	20	>7 × 10^8^			
39					
38	19	6.9			
37		3.4			
36	18	1.8			
35		1.0			
34	17	5.9 × 10^7^			
33		3.1	*s* = 8		
32	16	1.7	>7 × 10^6^		
31		1.0			
30	15	5.8 × 10^6^	6.9		
29		3.1	3.4		
28	14	1.7	1.8		
27		1.0	1.0		
26	13	5.8 × 10^5^	5.9 × 10^5^		
25		3.1	3.1	*s* = 6	
24	12	1.7	1.7	>7 × 10^4^	
23		1.0	1.0		
22	11	5.8 × 10^4^	5.8 × 10^4^	6.9	
21		3.1	3.1	3.4	
20	10	1.7	1.7	1.8	
19		1.0	1.0	1.0	
18	9	5.8 × 10^3^	5.8 × 10^3^	5.9 × 10^3^	
17		3.1	3.1	3.1	*s* = 4
16	8	1.7	1.7	1.7	>7 × 10^2^
15		1.0	1.0	1.0	
14	7	5.8 × 10^2^	5.8 × 10^2^	5.8 × 10^2^	6.9
13		3.1	3.1	3.1	3.4
12	6	1.7	1.7	1.7	1.8
11		1.0	1.0	1.0	1.0
10	5	5.8 × 10^1^	5.8 × 10^1^	5.8 × 10^1^	5.9 × 10^1^
9		3.1	3.1	3.1	3.1
8	4	1.7	1.7	1.7	1.7
7		1.0	1.0	1.0	1.0
6	3	5.8 × 1	5.8 × 1	5.8 × 1	5.8 × 1
5		3.1	3.1	3.1	3.1
4	2	1.7	1.7	1.7	1.7
3		1.0	1.0	1.0	1.0
2	1	0.6	0.6	0.6	0.6
1		<0.6	<0.6	<0.6	<0.6
0	0				
Approximate range		10^9^	10^7^	10^5^	10^3^
Factor, 95% Fiducial limits^b^		*n* = 2	6.6		
(X, ‐:‐)		*n* = 4	3.8		

^a^ Calculated from Table VIII_2_ of Fisher and Yates (1963).

^b^ Cochran; Biometrics (1950) 6: 105.
